# Emotional interference-based forgetting in short-term memory. Cognitive inhibition of pleasant but not unpleasant biologically relevant distractors

**DOI:** 10.3389/fpsyg.2015.00582

**Published:** 2015-05-06

**Authors:** Javier García-Pacios, David Del Río, Dolores Villalobos, José M. Ruiz-Vargas, Fernando Maestú

**Affiliations:** ^1^Department of Psychology, Faculty of Health Sciences, Camilo José Cela UniversityMadrid, Spain; ^2^Laboratory of Cognitive and Computational Neuroscience, Center for Biomedical Technology (Technical University of Madrid and Complutense University of Madrid)Madrid, Spain; ^3^Department of Basic Psychology II, Complutense University of MadridMadrid, Spain; ^4^Department of Basic Psychology, Autónoma University of MadridMadrid, Spain

**Keywords:** working memory, forgetting, emotional distraction, cognitive inhibition, interference

## Abstract

Emotional stimuli automatically recruit attentional resources. Although this usually brings more adaptive responses, it may suppose a disadvantage when emotional information is task-irrelevant and should be ignored. Previous studies have shown how emotional stimuli with a negative content exert a greater interference than neutral stimuli during a concurrent working memory (WM) task. However, the impact of positively valenced stimuli as interference has not been addressed to date. In three experiments and one re-analysis we explore the impact of pleasant and unpleasant emotional distractors during WM maintenance. The results suggest that our cognitive control can cope with the interference posed by pleasant distractors as well as with the interference posed by neutral stimuli. However, unpleasant distractors are harder to control in the context of WM maintenance. As unpleasant stimuli usually convey relevant information that we should not to ignore, our executive control seems to be less able to reallocate cognitive resources after unpleasant distraction.

## Introduction

The effect of emotion on our cognition and behavior is an issue widely addressed by the psychological literature. The wealthy interactions between these “hot” and “cold” systems have attracted widespread attention. In particular, interactions between memory and emotion are particularly interesting due to the opposing consequences seen when emotional information is a relevant part of the current activity (Canli et al., 2000), contrary to when emotional facts are irrelevant. Emotional stimuli automatically fall into the focus of our attention (Mogg et al., 1997; Ohman et al., 2001; Armony and Dolan, 2002). Such an effect is explained by their biological relevance, since emotional stimuli contain information that is important for survival (e.g., food or predators) (LeDoux, 1996; Ohman et al., 2000; Anderson and Phelps, 2001). The concept of “motivated attention” (Lang et al., 1993, 1998b) proposes that emotional information seems to have a privileged access to our cognitive system, by recruiting attentional resources automatically and improving our preparation to process it (Lang et al., 1998; Morris et al., 1998; Bradley et al., 2003; Sabatinelli et al., 2005). This phenomenon usually brings more adaptive responses since we can easily and accurately remember information crucial to our survival. Many laboratory studies have reported enhanced memory for emotional pictures (Canli et al., 2000), emotional word-lists (Jones et al., 1987; Dietrich et al., 2001), or for humor (Schmidt and Williams, 2001). However, there are other situations in which the most adaptive behavior consists precisely in ignoring emotional information, for example to accomplish a more immediate goal. It is in these circumstances when the biological salience of emotional stimuli, and our natural predisposition to deeply process them, turn those events into powerful interferences that compete with relevant information for cognitive resources (Ellis and Ashbrook, 1988). This finally results in a worsening of performance of the current task (Dolcos and McCarthy, 2006; Dolcos et al., 2008; Anticevic et al., 2010; Chuah et al., 2010; Denkova et al., 2010).

Detrimental effects of emotional interference on working memory (WM) provide an opportunity to explore the limits of cognitive control in memory, taking into account that evolution has prepared us to pay attention to emotional stimuli (Lang et al., 1998; Morris et al., 1998; Bradley et al., 2003; Sabatinelli et al., 2005). Ignoring threatening stimuli, such as an approaching predator, might be detrimental to our survival. On the other side, task-irrelevant stimuli should be ignored in order to avoid interference effects in WM to perform many everyday tasks. Indeed, suppression of unpleasant information may be considered essential for our mental health. Dealing with negative intrusive thoughts, memories, and images are part of our daily life, and difficulties in their control are part of the core symptoms related to anxiety and depression (Küpper et al., 2014; Catarino et al., 2015). In such a scenario, being able to prevent attention towards emotional information constitutes the most adaptive response, particularly when emotional information is not relevant or even detrimental for to immediate goals.

To our knowledge, few studies have addressed the issue of interference-based forgetting in WM due to the appearance of emotional distractors. Dolcos and colleagues, for example, have conducted a series of fMRI studies using several modifications of the same WM task. In an early study (Dolcos and McCarthy, 2006), they used a delayed-recognition WM task with sets of three human faces as items to be memorized and pictures depicting unpleasant and neutral scenes, as well as digitally scrambled versions of these pictures, as distractors presented during the delay interval. The worst recognition scores were associated with the appearance of unpleasant distractors. These results confirm the idea previously introduced: emotional stimuli exerted a more powerful interference than non-emotional distractors in WM. However, in a later study using a similar task (Dolcos et al., 2008), they did not find any behavioral effect. Further exploration identified a subgroup of participants who seem to profit from emotional interference. Other studies from this group have been developed to investigate the effect of sleep deprivation (Chuah et al., 2010) and anxiety-induced distraction (Denkova et al., 2010) in WM. They replicated the main effect of worse WM performance after unpleasant distraction, than after non-emotional distraction.

In a similar vein, Anticevic et al. (2010) addressed this issue in a delayed-recognition WM task using complex geometric shapes as relevant items to memorize and recognize. During the maintenance stage, three types of distractors were presented: unpleasant emotional pictures, neutral pictures, and task-related geometric shapes. A fourth condition was added as non-interference, in which no distractor was introduced. The authors also manipulated the difficulty of the task by including trials in which either two or four geometric shapes were presented at the encoding stage. Consistent with previous work (Dolcos and McCarthy, 2006; Chuah et al., 2010; Denkova et al., 2010), unpleasant distractors were associated with a worsening of accuracy compared with neutral distractors in low WM load trials. In the high load condition, negative and neutral stimuli seemed equally disruptive. According to the authors, the lack of an effect for emotional interference under high load is explained because, as difficulty increases, the effect of negative emotional interference was not detectable and therefore all types of distraction may have been equally disruptive.

Hence, results from different studies from several groups suggest that unpleasant emotional irrelevant stimuli seem to worsen the maintenance of neutral relevant information in WM (Dolcos and McCarthy, 2006; Anticevic et al., 2010; Chuah et al., 2010), although this effect might not be very consistent, since in some other studies it has been found only in a subset of participants (Dolcos et al., 2008) or only within the most confident responses (Denkova et al., 2010). Furthermore, all these previous studies on the interaction of emotional interference and WM performance have left open many important issues on how we regulate emotional interference over WM information.

First, and critically, the effect of pleasant emotional interference in WM is still an open issue. Some authors have pointed out that stimuli with higher levels of arousal could recruit more attentional resources (Lang et al., 1998), disrupting performance when they are not relevant for the ongoing task. But, has the affective valence of those highly arousing stimuli any influence on their power as distractors? Both positively and negatively valenced stimuli seem to recruit attentional resources because of their importance for survival (Bradley et al., 2003) and are thus associated with higher arousal than neutral stimuli. In this regard, both pleasant and unpleasant stimuli have been shown to capture attention automatically (Keil and Ihssen, 2004; Keil et al., 2006; De Oca et al., 2012). However, positively and negatively valenced stimuli differ critically in their consequences for our survival: while unpleasant stimuli are associated with threatening and avoidance behaviors, pleasant stimuli enhance approaching behaviors. Both arousal and valence are key dimensions in emotional processing (Lang et al., 1998a). However, by using only emotionally unpleasant stimuli, previous studies are not able to tease apart the role of arousal and valence in emotional interference in WM. Exploring the effect of both pleasant and unpleasant interference in comparison with neutral interference might provide us with valuable information about the mechanisms that make this type of stimuli powerful sources of interference.

A second issue arising from previous studies is whether participants might be able or not to completely ignore neutral stimuli in comparison with emotional distractors. The impact of non-emotional interference in WM has been largely explored, leading to several interpretations of the interference-based forgetting in short-term memory (Nairne, 1990; Jolicoeur and Dell'Acqua, 1998; Farrell and Lewandowsky, 2002; Barrouillet et al., 2004; Wixted, 2004; Oberauer and Kliegl, 2006). According to these theories, most kinds of interfering stimulus might be able to disrupt to some extent WM performance, in comparison to a distraction-free scenario. But results from studies in emotional-based interference in WM have been inconclusive on this issue. As stated before, the first study by Dolcos and McCarthy (2006) showed a stronger effect of unpleasant stimuli on WM performance, followed by neutral and, finally, by scrambled pictures. Accuracy was also lower for neutral than for scrambled distractors. These results are in accordance with interference-based forgetting theories (Berman et al., 2009) since the introduction of any kind of distracting stimuli impaired the recovery of information in comparison with a non-meaningful scrambled picture. This might support the idea of a graded interference from non-meaningful to neutral and finally to emotional stimuli. But some other studies have not found differences when comparing the disruption exerted by scrambled pictures and neutral stimuli (Chuah et al., 2010; Denkova et al., 2010). According to these results, one possibility that cannot be excluded is that the content of neutral stimuli which are irrelevant for the WM task is blocked out from entering into WM and thus does not cause interference. The difference between the interference posed by neutral and emotional stimuli might then depend on whether that stimulus enters WM or not. In other words, emotional stimuli might automatically gain access, while neutral ones might be blocked out whenever they are not task-relevant.

One way of examining whether or not this is the case is to introduce a non-interference condition as well. Anticevic et al. (2010) added this non-interference condition to their experimental setting, in which no distractor was presented. Surprisingly, no differences appeared in high WM load and accuracy for the non-interference condition was lower than for all distraction conditions. The authors explained this unexpected pattern as an artifact of their experimental design. All the conditions were pseudo-randomly presented so that the non-interference condition appeared during more than three consecutive trials. This made distraction trials much more common than free-distraction trials and therefore volunteers may have been surprised by the recognition stimulus on the non-interference trials. This hypothesis was supported by additional data using the same task but in which free-distraction trials were presented in a separate block, instead of intermixed with distraction trials (Anticevic et al., 2010). Using this blocked design, performance after non-interference was substantially better than after distraction conditions.

Other way to better understand if the difference between emotional and non-emotional distraction depends on whether or not they are blocked out from entering into WM would be to ensure that participants pay attention to both kinds of distracting stimuli. For instance, volunteers might be asked to respond to a question about the distracting stimuli. This would compel emotional and non-emotional stimuli to be attended, and thus any difference in their distracting power on the main WM task would not be attributable to an absence of attention allocated to neutral stimuli, but to the higher engagement caused by emotionally relevant stimuli.

Hence, the aim of the present work was to investigate how valence and arousal contribute to the interference posed by emotionally stimuli. Therefore, we used not only unpleasant, but also pleasant pictures as distractors in the context of a WM task. Based on the concept of motivated attention, we expected both kinds of emotional stimuli to automatically recruit attentional resources and disrupt WM performance to a higher extent than neutral stimuli do. Also, we aimed to clarify whether the difference between neutral and emotional stimuli arises because neutral stimuli are blocked out of attention while emotional stimuli are not, or because the latter are processed on a deeper level than the former once they all have access WM. If the difference between emotional and non-emotional interference comes from blocking non-emotional distractors out of attention, we expected performance when facing non-emotional interference to be indistinguishable from a non-interference scenario. Further, if the differential disruption posed by emotional distraction arises because of a lack of attention paid to neutral stimuli, the differences between emotional and non-emotional stimuli should vanish when people are explicitly asked to pay attention to any type of distractor.

We addressed these issues through three interrelated experiments and a re-analysis of the data from these experiments, using a WM task in which neutral faces were task-relevant items and emotional and non-emotional pictures were task-irrelevant distractors. In the first experiment we explored the mechanisms that make emotional stimuli powerful interferences. To do this, we included three experimental conditions in which pleasant, neutral, and unpleasant pictures were displayed as interference during the maintenance stage of the WM task. We aimed to verify whether pleasant interference has the same detrimental effect that unpleasant distractors have shown. Thus, contributions of both valence and arousal dimensions were investigated. In the second experiment, we further explored potential differences in the effects of emotional and non-emotional distraction in comparison with a non-interference scenario by adding a fourth condition to the original design, in which no stimulus was presented during the maintenance of task-relevant information. In the last experiment, we controlled the actual attentional engagement of our participants across conditions in order to discard potential differences in attentional capture that might be affecting the WM performance. Finally, we re-analyzed data from Experiments 2 and 3 to account for potential contributions of the arousal to the power of emotional stimuli as distractors in WM.

## Experiment 1

In the first experiment, we explored the effect of two valenced emotional distractors, pleasant, unpleasant, as well as the effect of neutral distraction in WM maintenance. If detrimental effects of unpleasant distraction were due to the biological relevance of emotional stimuli, other types of biologically relevant stimuli (i.e., pleasant events) should affect performance in a similar way. If this were the case, the worsening of performance by emotional distraction would seem to be mainly arousal-driven. Indeed, taking into account that pleasant stimuli are usually more arousing than neutral ones, but less arousing than unpleasant stimuli, performance after pleasant distraction should be better than after unpleasant distraction, but worse than after neutral distraction.

### Method

#### Participants

Participants were 30 students from the Complutense University of Madrid and the Camilo José Cela University of Madrid (mean age 21 year and a range between 18 and 35 years). They had normal or corrected-to-normal vision. The experiment was approved by the institutional Review Committee of the Center for Biomedical Technology (Technical University of Madrid and Complutense University of Madrid) and the procedure was performed in accordance with approved guidelines and regulations. This approval also covered the following experiments reported here. Half of the participants were females (18–35 years old and a mean age of 19.46 years) and half of them were males (18–34 years old and a mean age of 22.66 years). They all completed the Spanish version of the Spielberger State-Trait Anxiety Inventory for Adults (Spielberger et al., 2002) and the Beck Depression Inventory (Beck et al., 2006) (see Table 1 for demographic information). Participants received course credits for their time.

**Table 1:** **Volunteer's demographic information in Experiment 1, 2, and 3**.

	**Age**	**STAI-S**	**STAI-T**	**BDI**
**EXPERIMENT 1**				
Mean	21.06	16.50	17.33	5.40
SD	5.00	8.26	8.50	4.28
**EXPERIMENT 2**				
Mean	21.69	15.41	16.86	6.81
SD	4.48	6.07	8.76	5.66
**EXPERIMENT 3**				
Mean	21.23	17.04	19.2	9.30
SD	2.62	9.10	11.70	6.22

*STAI-S, Spielberger State-Trait Anxiety Inventory for Adults—State score; STAI-S, Spielberger State-Trait Anxiety Inventory for Adults—Trait score; BDI, Beck Depression Inventory*.

#### Materials

Items at encoding and recognition stages consisted of colored images of neutral faces. An oval mask was applied along the contours of the faces to remove ears and hair and avoid any potential non-face specific cues. A pair of faces was presented at the encoding stage while just one face was displayed at the recognition stage. Faces were counterbalanced across experimental conditions. For the distracting items presented at the maintenance period, 90 pictures from the International Affective Picture System (IAPS) (Lang et al., 2005) were selected and matched in luminance, contrast, color, and figure-ground relationships. They were divided into three experimental sets according to their normative valence and arousal ratings: pleasant, neutral, and unpleasant pictures (see Table 2 for mean normative values).

**Table 2:** **Mean normative values of pictures used in Experiment 1, 2, and 3, and mean subjective ratings of those pictures by our volunteers**.

**Condition**	**IAPS Valence**	**IAPS Arousal**	**Subjective Valence**	**Subjective Arousal**
**EXPERIMENT 1**				
Pleasant	7.33 (0.33)	5.84 (0.33)	7.14 (0.52)	5.35 (1.20)
Neutral	4.91 (0.35)	2.77 (0.35)	5.09 (0.52)	2.27 (0.50)
Unpleasant	2.29 (0.70)	6.54 (0.70)	2.23 (0.82)	6.48 (0.48)
**EXPERIMENT 2**				
Pleasant	7.34 (0.32)	6.23 (0.53)	7.09 (0.46)	5.40 (1.01)
Neutral	4.91 (0.35)	2.77 (0.38)	5.09 (0.55)	1.92 (0,66)
Unpleasant	2.39 (0.67)	6.23 (0.56)	2.37 (0.97)	6.71 (0.91)
**EXPERIMENT 3**				
Pleasant	7.34 (0.32)	6.23 (0.53)	7.48 (0.96)	5.86 (1.69)
Neutral	4.91 (0.35)	2.77 (0.38)	5.05 (0.62)	3.10 (1.4)
Unpleasant	2.39 (0.67)	6.23 (0.56)	2.40 (1.02)	6.261 (1.49)

*Standard deviations are showed in parenthesis*.

#### Procedure

**Figure 1:**
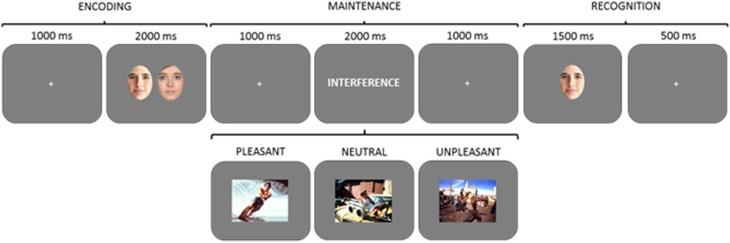
**Diagram of the delayed-recognition WM paradigm in Experiment 1**. Three types of distractors (pleasant, neutral, and unpleasant) were pseudorandomly presented during the maintenance stage. Volunteers were trained to learn and maintain the pair of faces into WM, look at the distractor, and then decide whether the face at the recognition stage was one of the two previously encoded or not, by pressing one of two keys.

A delayed-recognition WM paradigm with three experimental conditions, pleasant, neutral and unpleasant distraction was used. Each condition comprised 30 trials. Each trial began with a 1000 ms intertrial interval (ITI), followed by the presentation of a pair of faces for 2000 ms (encoding phase). After a 1000 ms blank screen, an interfering stimulus was displayed for 2000 ms, followed by another 1000 ms blank screen (maintenance phase). Next, just one face appeared on the screen for 1500 ms, followed by a 500 ms blank screen (recognition stage). Participants had to decide whether the face at the recognition stage had been one of the two previously encoded or not, by pressing one of two keys (Figure 1). Before the experiment, all of the volunteers underwent four training trials in order to ensure that they completely understood the task. To avoid inducing long-lasting mood states, the order of trials were constrained so that no more than three trials of the same condition were consecutively presented. Once the WM paradigm was completed, all the pictures used as distractors were presented to the participants and they were asked to rate them regarding emotional valence and arousal, using the Self-Assessment Manikin (SAM) self-report scale (Lang, 1980). Participants were allowed to see each picture for as long as they wanted. The order of presentation of the pictures was also constrained in the same way, although in a different sequence, as the WM task.

### Results

#### Accuracy

**Figure 2:**
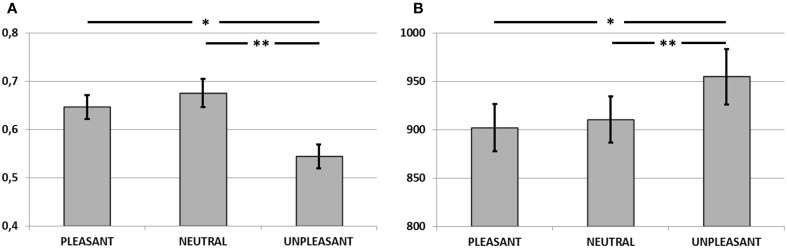
**(A)** Corrected recognition scores (hit rate—false alarm rate) in Experiment 1. Unpleasant distractors caused a detrimental effect on WM accuracy, compared to neutral and pleasant distractors (^*^p < 0.005; ^**^p < 0.001). **(B)** Mean reaction times for accurate recognitions in Experiment 1. Unpleasant distractors caused a slower performance on WM, compared to neutral and pleasant distractors (^*^p < 0.005; ^**^p < 0.0001). Error bars represent standard error of mean.

Figure 2A plots the corrected recognition score (hit rate—false alarm rate) for each condition, averaged across participants. A One-Way repeated-measures analysis of variance (ANOVA) revealed a significant main effect of condition [*F*_(2, 28)_ = 9.60, *p* < 0.001, η^2^ = 0.40]. Pairwise comparisons revealed a lower performance during unpleasant compared to pleasant (*p* < 0.005) and neutral distraction (*p* < 0.001). There were no differences between pleasant and neutral distraction (*p* > 0.1). These results were confirmed when statistical analyses were computed on the *d'*-values estimated for each condition [*F*_(2, 28)_ = 11.19, *p* < 0.0001, η^2^ = 0.44; pleasant > unpleasant (*p* < 0.005); neutral > unpleasant (*p* < 0.0001); pleasant = neutral (*p* > 0.1)].

#### Reaction times

Figure 2B shows the mean reaction times for correctly recognized items for each condition. Results from One-Way repeated-measures ANOVA yielded a main effect of condition [*F*_(2, 28)_ = 11.87, *p* < 0.001, η^2^ = 0.45]. Pairwise comparisons revealed slower performance during unpleasant compared to pleasant (*p* < 0.0001) and neutral distraction (*p* < 0.005). No differences were found between pleasant and neutral distraction (*p* > 0.1).

#### Subjective emotional ratings

As expected, subjective valence ratings differed as a function of affective category [*F*_(2, 28)_ = 284.85, *p* < 0.0001, η^2^ = 0.95], with pleasant pictures rated as most pleasant followed by neutral pictures, and unpleasant pictures rated as least pleasant (*p* < 0.0001 for all comparisons). Arousal ratings also varied as a function of affective category [*F*_(2, 28)_ = 139.47, *p* < 0.0001, η^2^ = 0.90], with pleasant and unpleasant pictures rated as more arousing than neutral pictures (*p* < 0.0001 for both comparisons). Unpleasant pictures were rated as more arousing than pleasant pictures (*p* <.0001) (see Table 2 for mean normative values and mean subjective values).

#### Item analysis

To test the relationship between the emotional features of pictures and their value as distractors during the WM task, we averaged the recognition and reaction time for each trial in which they appeared across participants. Then, we calculated the correlation between these measures and the mean valence and arousal subjective rating for each distractor. Valence correlated positively with accuracy [ρ_s(88)_ = 0.22, *p* < 0.05] and negatively with reaction time [ρ_s(88)_ = −0.37, *p* < 0.001], while arousal correlated positively with reaction time [ρ_s(88)_ = 0.22, *p* < 0.05], but did not with accuracy [ρ_s(88)_ = −0.16, *p* > 0.1]. Since both pleasant and unpleasant stimuli were highly arousing, partial correlations were calculated in order to test whether one of them was leading the correlation effect. Valence correlated positively with accuracy (ρvalence, accuracy · arousal = 0.21, *p* < 0.05) and negatively with reaction time (ρvalence, accuracy · arousal = −0.30, *p* < 0.005) when the effect of arousal was controlled. No significant correlation was found between arousal and accuracy (ρvalence, accuracy · arousal = −0.12, *p* > 0.1) nor between arousal and reaction time (ρ valence, accuracy · arousal = 0.11, *p* > 0.1) when the effect of valence was controlled.

### Discussion

One of the major aims of this first experiment was to clarify previous results suggesting that unpleasant emotional stimuli disrupt WM maintenance of non-emotional information more than neutral stimuli. In accordance to the literature (Dolcos and McCarthy, 2006; Dolcos et al., 2008; Anticevic et al., 2010; Chuah et al., 2010; Denkova et al., 2010) unpleasant distraction does affect WM more than neutral distraction, resulting in enhanced forgetting. Analysis of reaction times for correct responses also showed this pattern, with slower responses after unpleasant than after neutral distraction. This suggests that unpleasant interference increases the probability of forgetting and produces higher cognitive costs even for successful performance. This effect may be explained under the concept of motivated attention (Bradley et al., 2003) which refers to the automatic attentional resources captured by those stimuli that represent information linked to survival. As posed above, this capture of attentional resources means an advantage when emotion is task relevant, since it drives a deeper and more effective processing of those stimuli (Bradley et al., 2003). However, when emotion is not task relevant, this attentional capture and the following preferential processing of the attended information turns emotional stimuli into powerful interferences that compete with relevant information for cognitive resources. This finally worsens performance of the ongoing task (Dolcos and McCarthy, 2006; Dolcos et al., 2008; Anticevic et al., 2010; Chuah et al., 2010; Denkova et al., 2010).

A second major aim of this experiment was to investigate whether pleasant distraction affects WM in a similar manner to unpleasant distraction. If we assume that emotional features of stimuli turn them into powerful interferences, pleasant stimuli, which also represent biologically relevant information, such as food or reproduction, should also recruit more attentional resources and, therefore, should also be preferentially processed. Thus, they should compete with relevant information for attentional resources and they should produce a similar amount of forgetting for such information. Unexpectedly, pleasant distraction does not affect maintenance of information in WM more than neutral distraction. These results suggest that, contrary to our initial hypothesis, the power of emotional stimuli as interference in WM is not only driven by arousal. If this were the case, pleasant distractors would have produced lower WM performance than neutral distractors, but higher WM performance than unpleasant distraction. As this was not the case, the valence of emotional stimuli must contribute to their value as distractors. Partial correlation analysis from our data confirmed this hypothesis. Valence correlated significantly with performance when the effect of arousal was controlled, so that the more unpleasant the distractor was, the higher probability of forgetting the previously encoded information. Also, higher levels of unpleasantness in the stimuli predicted higher cognitive costs for correct responses, as reflected by reaction times.

Previous studies in the attentional blink phenomenon have showed that both pleasant and unpleasant stimuli equally capture more attention than neutral stimuli (Keil and Ihssen, 2004; Keil et al., 2006; De Oca et al., 2012). Thus, it seems not very probable that, in our study, differences in WM performance between pleasant and unpleasant distraction were due to a higher attentional capture by unpleasant stimuli, when compared with pleasant distractors. However, our executive control, specifically our inhibitory control, may not be equally capable of reallocating cognitive resources after the initial attentional response elicited by pleasant and by unpleasant distractors. Unpleasant stimuli convey important biological information that the brain has learned not to ignore, such as information related to threatening events. Therefore, it seems reasonable that such reallocation of cognitive resources towards the memory maintenance of the previously encoded relevant information was weaker after unpleasant distractors than after another kind of biologically relevant, but not threatening stimuli (Dolcos and McCarthy, 2006; Dolcos et al., 2008; Anticevic et al., 2010; Chuah et al., 2010; Denkova et al., 2010).

## Experiment 2

In the second experiment we first tried to confirm the unexpected finding of equivalent performance after pleasant and neutral distraction showed in Experiment 1. Second, we adjusted the selection of distractors in order to make pleasant and unpleasant conditions equal in arousal. Finally, we attempted to reveal potential differences in the effect of emotional and non-emotional distraction in comparison to a no-distraction scenario. As previously stated, the comparison of a neutral and a free distraction condition might help us disentangle whether neutral pictures are also posing a significant interference in WM or not.

### Method

#### Participants

Participants were 43 students from the Complutense University of Madrid and the Camilo José Cela University of Madrid (mean age 21.6 years; range from 18 to 40 years). They had normal or corrected-to-normal vision. 24 participants were females (18–33 years old and a mean age of 21.7 years) and 19 were males (18–40 years old and a mean age of 21.6 years). They all completed the Spanish version of the Spielberger State-Trait Anxiety Inventory for Adults (Spielberger et al., 2002) and the Beck Depression Inventory (Beck et al., 2006) (see Table 1 for demographic information).

#### Materials

Items at encoding and recognition were exactly the same as those used in Experiment 1(see Materials in Experiment 1), and they were also counterbalanced across experimental conditions. For the distracting items presented at the maintenance period, 90 pictures from the IAPS (Lang et al., 2005) were selected and matched in luminance, contrast, color and figure-ground relationships. They were divided into pleasant, neutral and unpleasant pictures. For this experiment we adjusted the criterion of selection to insure that pleasant and unpleasant conditions were equal in arousal (see Table 2 for mean normative values).

#### Procedure

**Figure 3:**
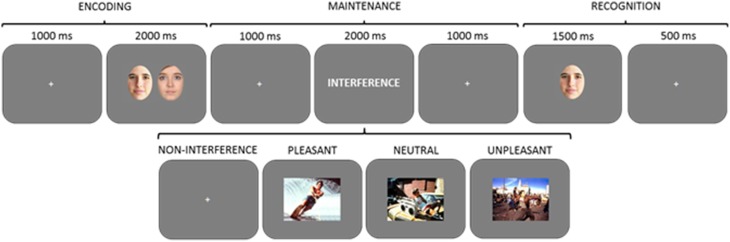
**Diagram of the delayed-recognition WM paradigm in Experiment 2 and 3**. Three types of distractors (pleasant, neutral, and unpleasant) were pseudorandomly presented during the maintenance stage. Volunteers were trained to learn and maintain the pair of faces into WM, look at the distractor, and then decide whether the face at the recognition stage was one of the two previously encoded or not, by pressing one of two keys. In a separate block, volunteers performed a fourth experimental condition with a maintenance period free of distraction.

A delayed-recognition WM paradigm with four experimental conditions no-distraction, pleasant, neutral, and unpleasant distraction was used and all conditions comprised 30 trials. The trial structure, times of presentation and instructions were the same as those used in Experiment 1. The order of trials was also constrained in the same way as in Experiment 1 (see Procedure in Experiment 1). However, no-distraction trials were presented in a separate block to avoid potential experimental artifacts. Although a blocked presentation of the no-distraction condition may be considered a methodological inconvenience, results from previous studies have shown that it is a suitable approach to prevent experimental artifacts that have already been reported, and that may affect performance in WM maintenance after no-distraction (Anticevic et al., 2010). That is, if presentation of all of the 120 trials would have been intermixed, distraction trials would have been much more common than no-distraction trials. In addition, the period of time without any visual stimulation during the maintenance stage was would have been much longer in no-distraction (4 s) than in distraction trials (1 s at the most) (see Figure 3). Therefore, volunteers might have been surprised by the appearance of a no-distraction trial. This could produce a worsening in performance for this condition not related to the processes we are interested in (Anticevic et al., 2010). Additionally, the order of presentation of no-distraction and interference blocks was counterbalanced across participants in order to eliminate any potential practice or fatigue effect.

### Results

#### Accuracy

**Figure 4:**
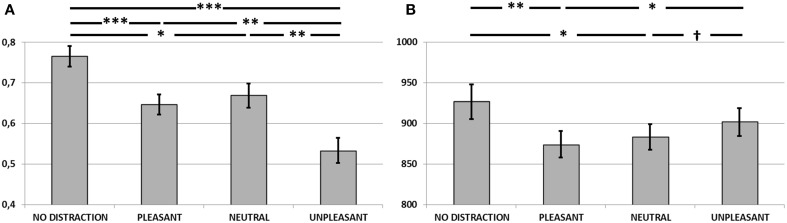
**(A)** Corrected recognition scores (hit rate—false alarm rate) in Experiment 2. Unpleasant distractors caused a detrimental effect on WM accuracy, compared to neutral and pleasant distractors, as well as to a scenario free of distraction (^*^p < 0.005; ^**^p < 0.001; ^***^p < 0.0001). **(B)** Mean reaction times for accurate recognitions in Experiment 2. Pleasant distractors caused a faster performance on WM, compared to unpleasant distractors and a scenario free of distraction (^*^p < 0.05; ^**^p < 0.005). Performance during unpleasant distraction might also tend to be slower than during neutral distraction († *p* = 0.08). Error bars represent standard error of mean.

Figure 4A plots the corrected recognition score (hit rate—false alarm rate) for each condition, averaged across participants. A One-Way repeated-measures ANOVA revealed a significant main effect of condition [*F*_(3, 40)_ = 19.76, *p* < 0.0001, η^2^ = 0.59]. Pairwise comparisons showed lower performance during unpleasant distraction compared to no-distraction (*p* < 0.0001), pleasant (*p* < 0.001) and neutral distraction (*p* < 0.001). Performance during pleasant and neutral distraction was also worse than during no-distraction (*p* < 0.0001; and *p* < 0.005, respectively). No differences were found between pleasant and neutral distraction (*p* > 0.1). These results were confirmed when statistical analysis were computed on the *d'*-values estimated for each condition [*F*_(3, 40)_ = 16.23, *p* < 0.0001, η^2^ = 0.54; no-distraction > pleasant (*p* < 0.0001), neutral (*p* < 0.005) and unpleasant (*p* < 0.0001); pleasant > unpleasant (*p* < 0.0001); neutral > unpleasant (*p* < 0.0001); pleasant = neutral (*p* > 0.1)].

#### Reaction times

Figure 4B shows mean reaction times for correctly recognized items in each condition. Results from One-Way repeated-measures ANOVA yielded a main effect of condition [*F*_(3, 40)_ = 5.34, *p* < 0.005, η^2^ = 0.28]. Pairwise comparisons showed faster performance during pleasant compared to no-distraction (*p* < 0.005) and unpleasant distraction (*p* < 0.05). Results from *post-hoc* comparisons also showed a faster performance during neutral than during no-distraction scenario (*p* < 0.05). Although not significant, our volunteers tended to respond slower after unpleasant than after neutral distraction (*p* = 0.08). There were no differences between pleasant and neutral distraction (*p* > 0.1), nor between unpleasant and no-distraction (*p* > 0.1).

#### Subjective emotional ratings

As expected, subjective valence ratings differed as a function of affective category [*F*_(2, 41)_ = 239.18, *p* < 0.0001, η^2^ = 0.92], with pleasant pictures rated as most pleasant followed by neutral pictures, and unpleasant pictures rated as least pleasant (*p* < 0.0001). Arousal ratings also varied as a function of affective category [*F*_(2, 41)_ = 162.04, *p* < 0.0001, η^2^ = 0.88], with pleasant and unpleasant pictures rated as more arousing than neutral pictures (*p* < 0.0001 for all comparisons). Although both pleasant and unpleasant pictures were selected to be equal in arousal, our volunteers rated, on average, unpleasant pictures as more arousing than pleasant pictures (*p* < 0.0001) (see Table 2 for mean normative values and mean subjective values).

#### Item analysis

As we did in Experiment 1, we tested the relationship between the emotional features of pictures and their value as distractors during the WM task. To do this, we calculated the correlation between their valence and arousal subjective ratings, and the averaged recognition and reaction time for each trial in which they appeared. Valence correlated positively with accuracy [ρ_s(88)_ = 0.23, *p* < 0.05] and negatively with reaction time [ρ_s(88)_ = −0.27, *p* < 0.01], while arousal tended to correlate negatively with accuracy [ρ_s(88)_ = −20, *p* = 0.05] and positively with reaction time [ρ_s(88)_ = −0.25, *p* < 0.05]. As previously mentioned, although we adjusted the criterion of selection to keep pleasant and unpleasant conditions equal in arousal, our volunteers rated unpleasant distractors as more arousing than pleasant ones. Therefore, partial correlations were also calculated in order to disentangle the effects of valence from the effects of arousal. In line with the Experiment 1, the positive correlation between valence and accuracy was marginally significant (ρ valence, accuracy · arousal = 0.19, *p* = 0.06) when the effect of arousal was controlled. Valence also correlated negatively with reaction time (ρ valence, accuracy · arousal = −0.26, *p* < 0.05) when the effect of arousal was controlled. Again, no significant correlation was found between arousal and accuracy (ρ valence, accuracy · arousal = −0.13, *p* > 0.1) when the effect of valence was controlled, while arousal tended to positively correlate with reaction time (ρ valence, accuracy · arousal = 0.19, *p* = 0.06).

### Discussion

In accordance with the first experiment and previous literature, the highest forgetting occurs after unpleasant distraction, extending previous evidence to show that unpleasant events can work as powerful interferences for WM maintenance (Dolcos and McCarthy, 2006; Dolcos et al., 2008; Anticevic et al., 2010; Chuah et al., 2010; Denkova et al., 2010). As in Experiment 1, pleasant distraction does not affect WM more than neutral distraction. In this regard, although we equated pleasant and unpleasant pictures in arousal, our volunteers rated unpleasant pictures as more arousing than pleasant ones. In principle, one may argue that this fact might account for their differences in performance. However, if the effect of emotional distractors in WM were exclusively due to the arousal value, performance after pleasant distraction should have been worse than after neutral distraction, since pleasant pictures were rated as more arousing than neutral stimuli. But this was not the case, as both pleasant and neutral stimuli showed similar levels of interference. Furthermore, results from partial correlations between subjective valence and accuracy, blocking the effect of arousal, and between subjective arousal and accuracy, blocking the effect of valence, showed a greater contribution of valence to the power of emotional stimuli as distractors. This general effect resembles the one observed in the first experiment, so that the more unpleasant a distractor is perceived, the higher the probability of forgetting the information previously encoded.

Additionally, neutral distraction did lead to a higher forgetting than no-distraction, in accordance with previous results employing similar tasks (Dolcos and McCarthy, 2006; Anticevic et al., 2010). These findings provide further evidence in favor of the detrimental effect of both emotional and non-emotional distractors in WM and strengthen the interference-based forgetting theories (Berman et al., 2009).

Results from reaction times were not as straightforward as in Experiment 1. However, they are in accordance with them, suggesting that unpleasant distraction might produce higher cognitive cost even for successful performance. In addition, higher reactions times were recorded after no-distraction in WM maintenance. This might be motivated by the duration of the maintenance stage without any stimuli (4 s in this condition vs. 1 s for the others). The task was also much easier when no-distraction was presented (accuracy raised almost to 90% in this condition). Hence, our volunteers might have experienced a decrease in their concentration, leading to slower (yet more accurate) responses to probes.

## Experiment 3

Experiments 1 and 2 have consistently shown that people can cope with pleasant distraction as well as with neutral ones, while the presence of unpleasant irrelevant events negatively affect their WM performance. Those findings suggest that the level of the arousal in a distracting stimulus is not the primary dimension that makes it difficult to control. Rather, the valence of the stimulus appears to contribute most to its power as an interference. Our partial correlation analysis in both experiments supported this rationale, as the valence of distractors correlated with accuracy in the WM task, when the contribution of arousal was blocked. In contrast, arousal levels in the distractor did not predict WM performance when the contribution of valence was controlled. Even so, it is still possible that differences in accuracy and results from partial correlations would be reflecting an attentional bias rather than a cognitive control effect. That is, our volunteers might have paid less attention to neutral and pleasant distractors than to unpleasant distractors, and therefore the former would have interfered less than the latter, what would finally explain the behavioral differences in the WM task. Behavioral and psychophysiological studies in the attentional blink phenomenon (Keil and Ihssen, 2004; Keil et al., 2006; De Oca et al., 2012) have shown that pleasant stimuli automatically capture attention more than neutral events, and that they do so as much as unpleasant stimuli do. However, we cannot be sure about the actual attentional engagement of our participants across conditions, since we do not have any direct measure of that in Experiment 1 and 2. A suitable manner to answer this open issue would be to make participants explicitly assess each distractor. Therefore, in Experiment 3 we used exactly the same WM task that we employed in Experiment 2, but we asked volunteers to decide whether the scene represented in every single distractor took place indoors or outdoors, and to report it by pressing one of two keys. Asking participants to make a decision about something not related to the emotional features of the stimuli provided us with information about their attentional engagement, avoiding potential changes in the interfering power of pictures derived from an emotional re-evaluation of the event (Lazarus and Alfert, 1964; Gross and John, 2003). If a participant's judgments were accurate and did not show reliable differences across conditions, we could consider that they voluntarily paid full attention to the distractors, regardless of their emotional valence. If this were the case, and if the behavioral pattern of recognition in the WM task was the same as that observed in Experiment 1 and 2, we could discard the possibility that our differences in performance were due to differences in the amount of attention recruited by the three types of distractors.

### Method

#### Participants

Participants were 26 students from the Camilo José Cela University of Madrid (mean age 21.2 years; range from 18 to 28 years). They had normal or corrected-to-normal vision. 20 participants were females (18–28 years old and a mean age of 21.3 years) and 6 were males (18–24 years old and a mean age of 21 years). They all completed the Spanish version of the Spielberger State-Trait Anxiety Inventory for Adults (Spielberger et al., 2002) and the Beck Depression Inventory (Beck et al., 2006) (see Table 1 for demographic information). Volunteers received course credits for their time.

#### Materials

Items at encoding and recognition were exactly the same ones as those used in Experiment 1 and 2 (see Materials in Experiment 1 and 2), and they were also counterbalanced across experimental conditions. The interfering items presented during the maintenance period were the same as those we used in Experiment 2 (Lang et al., 2005), and were therefore matched in luminance, contrast, color, and figure-ground relationships. They were also divided into pleasant, neutral, and unpleasant pictures (see Table 2 for mean normative values).

#### Procedure

The delayed-recognition WM paradigm was basically the same than that used in Experiment 2, four experimental conditions no-distraction, pleasant, neutral, and unpleasant distractions were also used, all of which comprised 30 trials. The trial structure, times of presentation, instructions and pseudorandomization of trials were the same as those used in Experiment 2. No-distraction trials were also presented in a separate block to avoid potential experimental artifacts that have been previously reported (Anticevic et al., 2010) (see Procedure in Experiment 2). However, in this experiment, volunteers had to respond whether the scene represented in the distracting picture occurred indoors or outdoors, and press one of two keys to report it. We avoided asking participants about the emotional features of the pictures, as we did not want them to re-assess their emotional content, as this might modify participant's emotional perception of the distractors, and therefore also modify their power as interferences (Lazarus and Alfert, 1964; Gross and John, 2003).

### Results

#### Indoors/outdoors judgments

Volunteers were highly engaged in the judgment of whether the distracting scene took place indoors or outdoors, with responses over 85% correct in all conditions. A One-Way repeated-measures ANOVA revealed no significant differences in accuracy across conditions [*F*_(2, 24)_ = 1.16, *p* > 0.1, η^2^ = 0.08].

#### Accuracy

**Figure 5:**
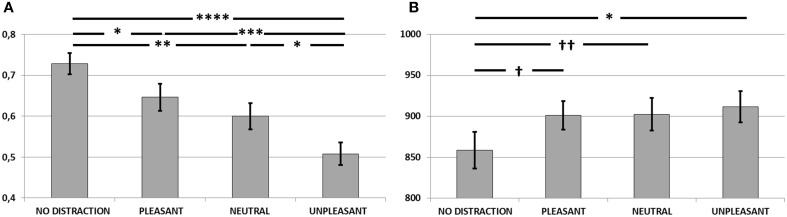
**(A)** Corrected recognition scores (hit rate—false alarm rate) in Experiment 3. Unpleasant distractors caused a detrimental effect on WM accuracy, compared to neutral and pleasant distractors, as well as to a scenario free of distraction (^*^p < 0.05; ^**^p < 0.005; ^***^p < 0.001; ^****^p < 0.0001). **(B)** Mean reaction times for accurate recognitions in Experiment 2. Unpleasant distractors caused a slower performance on WM, compared to a scenario free of distraction (^*^p < 0.05). Performance during pleasant and neutral distraction might also tend to be slower than during no-distraction († *p* = 0.07 and †† *p* = 0.08, respectively). Error bars represent standard error of mean.

Figure 5A plots the corrected recognition score (hit rate—false alarm rate) for each condition, averaged across participants. A One-Way repeated-measures ANOVA revealed a significant main effect of condition [*F*_(3, 23)_ = 16.16, *p* < 0.0001, η^2^ = 0.67]. Pairwise comparisons showed lower performance during unpleasant distraction compared to no-distraction (*p* < 0.0001), pleasant (*p* < 0.001) and neutral distraction (*p* < 0.05). Performance during pleasant and neutral distraction was also worse than during no-distraction (*p* < 0.05; and *p* < 0.005, respectively). No differences were found between pleasant and neutral distraction (*p* > 0.1). These results were confirmed when statistical analysis were computed on the *d'*-values estimated for each condition [*F*_(3, 23)_ = 12.21, *p* < 0.0001, η^2^ = 0.61; no-distraction > pleasant (*p* = 0.005), neutral (*p* = 0.005) and unpleasant (*p* < 0.0001); pleasant > unpleasant (*p* = 0.001); neutral > unpleasant (*p* < 0.05); pleasant = neutral (*p* > 0.1)].

#### Reaction times

Figure 5B shows mean reaction times for correctly recognized items in each condition. Results from One-Way repeated-measures ANOVA yielded a main effect of condition [*F*_(3, 23)_ = 3.25, *p* < 0.05, η^2^ = 0.11]. Pairwise comparisons showed slower performance during unpleasant distraction compared to no-distraction (*p* < 0.05) and a tendency towards slower performance after pleasant and neutral distraction than after no-distraction (*p* = 0.07 and *p* = 0.08, respectively). There were no differences between pleasant and neutral distraction (p > 0.1), between pleasant and unpleasant distraction (p > 0.1), nor between neutral and unpleasant distraction (p > 0.1).

#### Subjective emotional ratings

In line with Experiment 1 and 2, subjective valence ratings differed as a function of affective category [*F*_(2, 24)_ = 119.68, *p* < 0.0001, η^2^ = 0.90], with pleasant pictures rated as most pleasant followed by neutral pictures, and unpleasant pictures rated as least pleasant (*p* < 0.0001 for all comparisons). Arousal ratings also varied as a function of affective category [*F*_(2, 24)_ = 50.04, *p* < 0.0001, η^2^ = 0.80], with pleasant and unpleasant pictures rated as more arousing than neutral pictures (*p* < 0.0001 for all comparisons). As happened in Experiment 2, our volunteers rated, on average, unpleasant pictures as more arousing than pleasant pictures (*p* < 0.05), although both sets of pictures were selected to be equal in arousal (see Table 2 for mean normative values and mean subjective values).

#### Item analysis

In line with Experiment 1 and 2, we explored the relationship between the emotional features of pictures and their value as interference during the WM task, by correlating valence and arousal subjective ratings with the averaged recognition and reaction time for each trial in which they appeared. Valence tended to correlate positively with accuracy [ρ_s(88)_ = 0.23, *p* < 0.05] and did not correlate with reaction time [ρ_s(88)_ = −0.19, *p* = 0.07]. Arousal did not correlate with accuracy [ρ_s(88)_ = 0.03, *p* > 0.1] nor with reaction time [ρ_s(88)_ = 0.06, *p* > 0.1]. Partial correlations were also calculated to disentangle the effects of valence from the effects of arousal. In accordance with Experiment 1 and 2, valence correlated positively with accuracy (ρ valence, accuracy · arousal = 0.22, *p* < 0.05) when the effect of arousal was controlled. Valence also tended to correlate negatively with reaction time (ρ valence, accuracy · arousal = −0.18, *p* = 0.08) when the effect of arousal was controlled. Again, no significant correlation was found between arousal and accuracy (ρ valence, accuracy · arousal = −0.01, *p* > 0.1) nor with reaction time (ρ valence, accuracy · arousal = 0.00, *p* > 0.1) when the effect of valence was controlled.

### Discussion

In Experiments 1 and 2, we replicated previous results in the literature, showing that unpleasant distracting events do affect the maintenance of non-emotional information in WM (Dolcos and McCarthy, 2006; Dolcos et al., 2008; Anticevic et al., 2010; Chuah et al., 2010; Denkova et al., 2010). Our results also revealed that pleasant distractors did not affect WM maintenance more than neutral distractors, suggesting that the valence dimension is the main feature that turns an emotional event into a powerful interference. Partial correlation analysis in both experiments further supported such rationale. However, one might still be concerned about how much attention our participants had voluntarily paid to each type of distractor. In other words, our volunteers might just have ignored the neutral and pleasant distracting pictures, when compared with the unpleasant stimuli. Behavioral and psychophysiological studies in the attentional blink phenomenon (Keil and Ihssen, 2004; Keil et al., 2006; De Oca et al., 2012) have reported that pleasant stimuli actually capture attention as much as unpleasant stimuli do, but even so, there was a considerable degree of uncertainty in our experiments regarding this issue, since we did not have any direct measure of the actual attentional engagement of our participants across conditions. In this last experiment, we controlled this matter by asking our participants to make an assessment of each distractor, within the WM task, and to inform about this evaluation by pressing one of two buttons. Our volunteers were highly successful at judging whether or not the distraction scene took place indoors or outdoors, which confirms that they truly paid attention to the distracting stimuli. More importantly, there were no differences in this measure between conditions, revealing that they initially processed all types of distractors to the same extent. This manipulation did not seem to affect overall performance, as the corrected recognition score in each condition was similar to those reported in Experiment 1 and 2. Interestingly enough, the overall WM performance in this experiment, with the potential effect of differential attentional bias controlled, resembled the pattern observed in our two first experiments. Although biologically relevant, pleasant distractors did not affect WM maintenance more than neutral and non-emotional events. Again, partial correlation analysis revealed a contribution of valence rather than of arousal, to the power of emotional stimuli as distractors. Also, higher forgetting after neutral distraction was observed, in comparison with a WM maintenance free of distraction, further supporting interference-based forgetting theories (Berman et al., 2009).

In summary, this third experiment provides further evidence regarding the differential effect of two types of emotional distraction, pleasant and unpleasant events, in WM. As participants equally paid attention to all distractors, these results suggest that unpleasant stimuli were more interfering due to a weaker reallocation of cognitive resources after the initial attentional response.

## Re-analysis of experiments 2 and 3

Despite the converging results from Experiments 1, 2, and 3, a possible limitation of the current study is in regard to the subjective arousal ratings of participants in Experiments 2 and 3. Although we selected pleasant and unpleasant pictures equal in arousal, based on their IAPS normative values, our volunteers rated pleasant pictures as less arousing than unpleasant stimuli, which might make it difficult to disentangle the effects of valence from the effects of arousal. However, in both experiments, results from partial correlations between these emotional dimensions and accuracy, blocking first the effect of arousal and then the effect of valence, point to valence as the primary dimension responsible for the power of emotional stimuli as distractors. Furthermore, if arousal instead of valence were the most contributory dimension, performance after pleasant distraction would have been worse than after neutral distraction, since pleasant stimuli were rated as more arousing than neutral pictures. Still, a partial contribution of arousal cannot be completely discarded in light of the present data. Thus, in this last experiment we reanalyzed the data from Experiments 2 and 3, in which we employed exactly the same distracting stimuli, to compare those volunteers that subjectively perceived pleasant and unpleasant distractors as equally arousing with those who rated the unpleasant distractors as more arousing than pleasant scenes. If arousal had not an effect in participant's performance in the WM task, as we suggest based on results from our three previous experiments, both groups of participants should show the same overall effect. However, if the lower performance after unpleasant distraction were explained by the fact that volunteers perceived them as more arousing than the pleasant stimuli, the group of participants that perceived both conditions equal in arousal should also show equivalent performance after both pleasant and unpleasant distraction. By contrast, the group of volunteers that rated unpleasant pictures as more arousing should still exhibit the greater WM impairment after unpleasant than after pleasant distractors.

### Method

### Participants, procedure, and materials

Data from all participants in Experiment 2 (*n* = 43) and 3 (*n* = 26) were employed in this experiment (total *n* = 69), as items at the encoding and recognition stages, and interfering scenes were exactly the same as those experiments.

First, for each participant, we computed a repeated measures *t*-test between subjective arousal rating for pleasant and for unpleasant distractors. Twenty three participants showed equivalent arousal rating between both conditions (*p* > 0.1) and were included in the Balanced Arousal Group. Forty participants showed higher arousal ratings for unpleasant than for pleasant distractors (*p* < 0.05) and therefore were included in the Unbalanced Arousal Group. Four participants who showed neither a robust difference nor a clear equivalent arousal rating (*p*-values between 0.05 and 0.1) were excluded from further analysis. Finally, two volunteers who rated pleasant scenes as more arousing than unpleasant stimuli were also excluded from the analysis in order to reduce noise in the analysis. Thus, a final sample of 63 participants took part in this re-analysis.

As the Unbalanced Arousal Group had almost twice the size of the Balanced Arousal Group, we also computed the analysis using a subsample of 23 participants from the biggest group. To do this, we randomly selected 23 participants from the whole Unbalanced Arousal Group and compared them with the 23 participants in the Balanced Arousal Group. In order to prevent an effect on the results due to the random down-sampling of the Unbalanced Arousal Group, we repeated this procedure three more times and computed the analysis using these three different randomly down-sampled groups (see Supplementary Materials).

### Results

#### Accuracy

**Figure 6:**
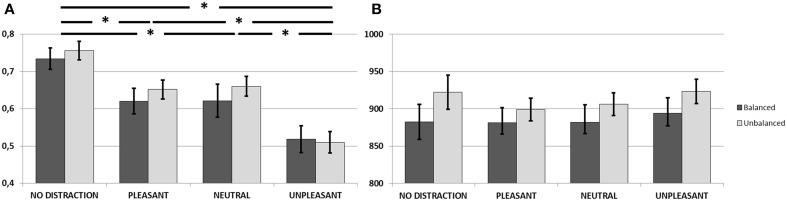
**(A)** Corrected recognition scores (hit rate—false alarm rate) in the Re-Analysis of Experiments 2 and 3. Unpleasant distractors caused a detrimental effect on WM accuracy, compared to neutral and pleasant distractors, as well as to a scenario free of distraction (^*^p < 0.0001). Neither the effect of Group nor the effect of interaction between Group and Condition were significant **(B)** Mean reaction times for accurate recognitions in Experiment 2. Neither the effect of Condition nor the effect of group nor the effect of interaction were significant. Error bars represent standard error of mean.

Figure 6A plots the corrected recognition score (hit rate–false alarm rate) for each group and condition, averaged across participants. A One-Way repeated-measures ANOVA with a between subjects factor revealed a significant main effect of condition [*F*_(3, 59)_ = 29.53, *p* < 0.0001, η^2^ = 0.60]. Pairwise comparisons showed lower performance during unpleasant distraction compared to no-distraction (*p* < 0.0001), pleasant (*p* < 0.0001) and neutral distraction (*p* < 0.0001). Performance during pleasant and neutral distraction was also worse than during no-distraction (*p* < 0.0001; and *p* < 0.0001, respectively). No differences were found between pleasant and neutral distraction (*p* > 0.1). Neither the effect of group [*F*_(1, 61)_ = 0.40, *p* > 0.1, η^2^ = 0.007] nor the effect of interaction [*F*_(3, 59)_ = 0.32, *p* > 0.1, η^2^ = 0.01] were significant.

These results were confirmed when statistical analysis were computed on the *d'*-values estimated for each condition. The main effect of condition was significant [*F*_(3, 59)_ = 21.96 *p* < 0.0001, η^2^ = 0.52; no-distraction > pleasant (*p* = 0.0001), neutral (*p* = 0.0001) and unpleasant (*p* < 0.0001); pleasant > unpleasant (*p* = 0.0001); neutral > unpleasant (*p* < 0.0001); pleasant = neutral (*p* > 0.1]. Again, neither the effect of group [*F*_(1, 61)_ = 0.83, *p* > 0.1, η^2^ = 0.01] nor the effect of interaction [*F*_(3, 59)_ = 0.86, *p* > 0.1, η^2^ = 0.04] were significant.

As the sizes of groups were notably different, and this might affect the results of the analysis, we repeated the analysis using a subsample of the biggest group, as previously described in the *Participants, Procedure, and Materials* section. Again the One-Way repeated-measures ANOVA with a between subjects factor revealed a significant main effect of condition [*F*_(3, 42)_ = 28.08, *p* < 0.0001, η^2^ = 0.66]. Pairwise comparisons also showed lower performance during unpleasant distraction compared to no-distraction (*p* < 0.0001), pleasant (*p* < 0.0001) and neutral distraction (*p* = 0.001). Performance during pleasant and neutral distraction was also worse than during no-distraction (*p* < 0.0001; and *p* < 0.0001, respectively). No differences were found between pleasant and neutral distraction (*p* > 0.1). In accordance with results on corrected recognition scores, neither the effect of group [*F*_(1, 44)_ = 0.14, *p* > 0.1, η^2^ = 0.01] nor the effect of interaction [*F*_(3, 42)_ = 0.14, *p* > 0.1, η^2^ = 0.01] were significant.

Results on *d*'-values also mirrored those on corrected recognition scores. The main effect of condition was significant [*F*_(3, 42)_ = 21.61 *p* < 0.0001, η^2^ = 0.60; no-distraction > pleasant (*p* < 0.0001), neutral (*p* < 0.0001) and unpleasant (*p* < 0.0001); pleasant > unpleasant (*p* < 0.0001); neutral > unpleasant (*p* = 0.001); pleasant = neutral (*p* > 0.1)]. Again, neither the effect of group [*F*_(1, 61)_ = 0.34, *p* > 0.1, η^2^ = 0.008] nor the effect of interaction [*F*_(3,42)_ = 0.35, *p* > 0.1, η^2^ = 0.02] were significant.

Although random, the actual down-sampling of the Unbalanced Arousal Group might still have had an effect in the analysis. To account for this potential problem, we performed three different random down-samplings and computed the same analysis on them. In each case, the results replicated the findings reported here, for both corrected recognition scores and *d'*-values (see Supplementary Materials).

#### Reaction times

Figure 6B shows mean reaction times for correctly recognized items in each group and condition. Results from a One-Way repeated-measures ANOVA with a between subjects factor did not show effects of condition [*F*_(3, 59)_ = 1.26, *p* > 0.1, η^2^ = 0.06], group [*F*_(1, 61)_ = 1.36, *p* > 0.1, η^2^ = 0.02] or interaction [*F*_(3, 59)_ = 0.20, *p* > 0.1, η^2^ = 0.01].

This analysis showed the same results when using a subsample of the biggest group, for the effect of condition [*F*_(3, 42)_ = 0.82, *p* > 0.1, η^2^ = 0.05], group [*F*_(1, 44)_ = 0.72, *p* > 0.1, η^2^ = 0.01] or interaction [*F*_(3, 42)_ = 0.36, *p* > 0.1, η^2^ = 0.02] (see also Supplementary Materials for results using different random down-samplings).

#### Subjective emotional ratings

As expected, subjective valence ratings differed as a function of affective category in both groups of participants [Balanced Arousal Group: *F*_(2, 21)_ = 72.08, *p* < 0.0001, η^2^ = 0.87; Unbalanced Arousal Group: *F*_(2, 38)_ = 365.56, *p* < 0.0001, η^2^ = 0.95], with pleasant pictures rated as most pleasant followed by neutral pictures, and unpleasant pictures rated as least pleasant (*p* < 0.0001 for all comparisons in both groups).

Arousal ratings also varied as a function of affective category [Balanced Arousal Group: *F*_(2, 21)_ = 46.05, *p* < 0.0001, η^2^ = 0.81; Unbalanced Arousal Group *F*_(2, 38)_ = 197.14, *p* < 0.0001, η^2^ = 0.91]. Both groups of participants rated pleasant and unpleasant pictures as more arousing than neutral pictures (*p* < 0.0001 for all comparisons in both groups) and, as expected, participants in the Balanced Arousal Group rated pleasant and unpleasant scenes as equally arousing (*p* > 0.1), while participants in the Unbalanced Arousal Group rated unpleasant pictures as more arousing than pleasant scenes (*p* < 0.0001).

Subjective emotional rating in the randomly down-sampled group mirrored those in the whole Unbalanced Arousal Group, with valence differing as a function of affective category [*F*_(2, 21)_ = 173.88, *p* < 0.0001, η^2^ = 0.94], with pleasant pictures rated as most pleasant followed by neutral pictures, and unpleasant pictures rated as least pleasant (*p* < 0.0001 for all comparisons), and arousal also differing as a function of affective category [Balanced Arousal Group: *F*_(2, 21)_ = 86.47, *p* < 0.0001, η^2^ = 0.89] with pleasant and unpleasant pictures rated as more arousing than neutral pictures (*p* < 0.0001 for all comparisons) (see Table 3 for mean normative values and mean subjective values). This pattern did not change in the three different random down-samplings (see Supplementary Materials).

**Table 3:** **Mean subjective ratings of pictures used in the Re-Analysis of Experiments 2 and 3**.

**Condition**	**Balanced group**		**Unbalanced group**		**Random down-sampling**	
	**Subjective valence**	**Subjective arousal**	**Subjective valence**	**Subjective arousal**	**Subjective valence**	**Subjective arousal**
Pleasant	7.40 (0.97)	5.88 (1.90)	7.10 (0.77)	5.26 (1.06)	7.13 (0.77)	5.49 (1.03)
Neutral	4.99 (0.44)	2.87 (1.42)	5.07 (0.45)	2.18 (1.13)	4.98 (0.46)	2.41 (1.32)
Unpleasant	2.77 (1.14)	6.07 (1.80)	2.24 (0.62)	7.00 (0.90)	2.16 (0.71)	7.08 (0.85)

*Standard deviations are showed in parenthesis*.

### Discussion

Experiments 1, 2, and 3 consistently showed that unpleasant distraction can impair the maintenance of non-emotional information in WM, while our executive system seems to be able to control pleasant distraction as well as neutral distraction. However, the fact that participants rated the unpleasant distractor as more arousing than the pleasant scenes is still a limitation in those experiments, especially in Experiments 2 and 3, where pleasant and unpleasant pictures were equivalent in arousal, based on their IAPS normative values. That is, our participants might have experienced a higher interference during the unpleasant distraction condition just because the scenes in that condition were more arousing than the scenes in the other two conditions. If this was true, one might also have expected worse performance after pleasant distraction than after neutral distraction, since pleasant scenes were rated as more arousing than neutral pictures, but this was not the case. Even though, we could not discard a potential contribution of arousal to the WM effect.

In this re-analysis we addressed this final concern by comparing those participants who perceived pleasant and unpleasant distractors equally arousing to those who rated the unpleasant distractors as more arousing than the pleasant scenes. Results from our re-analysis showed that the Unbalanced Arousal Group does show the same performance pattern that has been reported in Experiments 1, 2, and 3. More interestingly, participants in the Balanced Arousal group also exhibited lower WM accuracy after unpleasant distraction than after pleasant and neutral distraction. If the primary reason that the unpleasant scenes were more distracting was their higher level of perceived arousal, participants in this last group, who rated both pleasant and unpleasant scenes equal in arousal, should have experienced the same amount of interference during both types of distraction, but this was not the case. Moreover, our analysis did not reveal an effect of group and, critical for discarding a potential contribution of the arousal, they did not reveal an effect of interaction between group and condition. These results were also confirmed when the sample size of the groups was taken into account (see also Supplementary Materials).

In conclusion, results from this re-analysis support the findings reported in the three previous experiments while discarding a potential role of the arousal to the differences between pleasant and unpleasant scenes as distractors in WM.

## Discussion and conclusions

Few papers have addressed the effect of task-irrelevant emotional information during the maintenance of task-relevant non-emotional information, with the general finding that unpleasant distraction causes a negative effect in WM performance (Dolcos and McCarthy, 2006; Dolcos et al., 2008; Anticevic et al., 2010; Chuah et al., 2010; Denkova et al., 2010). Furthermore, the effect of pleasant distraction in WM remained unexplored. In four experiments we attempted to unravel the effect of both pleasant and unpleasant distractors in WM, when compared to non-emotional distractors, as well as to a scenario free of distraction. Results from all experiments confirmed the general finding that unpleasant distraction increases the probability of forgetting in short-term memory (Dolcos and McCarthy, 2006; Dolcos et al., 2008; Anticevic et al., 2010; Chuah et al., 2010; Denkova et al., 2010). This effect has been previously explained by the biological relevance of emotional stimuli. Provided that emotional stimuli represent crucial information for survival, our cognitive system tends to process them automatically (Bradley et al., 2003). In many occasions, this supposes an advantage since it helps us to exert more adaptive responses (Dolan, 2002) but when the most adaptive behavior entails ignoring emotional stimuli, they can compete with the maintenance of relevant information, worsening the performance of the ongoing task (Dolcos and McCarthy, 2006; Dolcos et al., 2008; Anticevic et al., 2010; Chuah et al., 2010; Denkova et al., 2010). According to this idea, pleasant emotional stimuli, which are also better remembered when they are task-relevant (Schmidt and Williams, 2001), should similarly worsen WM performance. Unexpectedly, results from Experiments 1 and 2 showed equivalent performance for neutral and pleasant distraction, suggesting that the valence might be the crucial dimension in an emotional-based interference effect. This was further supported by partial correlation analysis where the effect of arousal was controlled.

In light of these results, two different mechanisms may be proposed to account for differences in WM. First, unpleasant distractors may elicit a greater attentional response than neutral and pleasant distractors do, and therefore they may be processed more in depth, resulting in greater interference with the maintenance of relevant information. Although this may be true for neutral pictures, as it is well known that they recruit less attention than emotional events (Lang et al., 1998; Morris et al., 1998; Bradley et al., 2003; Sabatinelli et al., 2005), behavioral and psychophysiological studies have revealed that pleasant stimuli recruit as much attention as unpleasant stimuli do (Keil and Ihssen, 2004; Keil et al., 2006; De Oca et al., 2012). However, it is also possible that differences in WM performance between unpleasant and pleasant distractors were due to differences in the capacity of our executive control to reallocate cognitive resources after the initial attentional response. Unpleasant stimuli represent threatening information that is biologically important, so it is conceivable that the inhibition of the automatic processing of emotional information, in favor of the reallocation of resources to the ongoing task, was harder for unpleasant than for pleasant distractors. Thus, forgetting after unpleasant distraction may be more likely to happen than after pleasant or neutral distraction (Dolcos and McCarthy, 2006; Dolcos et al., 2008; Anticevic et al., 2010; Chuah et al., 2010; Denkova et al., 2010).

Even so, a lack of a direct measure of participant's attentional engagement across conditions left this question open. Although it seems improbable, our participants might voluntarily have been paying less attention to pleasant and neutral distractors than to unpleasant pictures and therefore, the processing of such information would have not even been initiated. Our manipulation in the third experiment, where we asked volunteers to pay attention to the distracting pictures and report their assessment, discarded that alternative explanation, as participants were highly and equally engaged across the three conditions. Since in these circumstances the pattern of WM performance did not vary from those observed in the Experiment 1 and 2, it can be argued that differences between pleasant and neutral distraction, when compared with unpleasant distraction are not due to a differential voluntary attentional engagement across conditions but to a differences in our cognitive capacity to override an automatic deep processing of the distractors.

Nevertheless, these three experiments left an open question, regarding a potential contribution of arousal to the reported WM effect, as overall, our participants perceived the unpleasant scenes as more arousing than the pleasant distractors, even when we selected both types of distractors to be equal in arousal, based on their IAPS normative values. To account for this final limitation, we performed a re-analysis to compare the WM performance of those participants who rated both types of distracting scenes equally arousing, to those who rated unpleasant pictures as more arousing than pleasant stimuli. Results from this re-analysis confirmed that both groups of participants did not differ in WM performance, and that both experienced higher interference during unpleasant distraction than after pleasant or neutral distraction.

Finally, previous studies in this field have addressed the effect of non-emotional distraction when compared to free-distraction maintenance. Although some of them found equivalent performance after neutral distraction and after non-interference maintenance (Chuah et al., 2010; Denkova et al., 2010), some others reported a better performance after no-distraction scenario than after neutral distraction (Dolcos and McCarthy, 2006; Anticevic et al., 2010). Our volunteers experience higher forgetting after neutral distraction than during a free-distractor maintenance period in both Experiment 2 and 3. This result provides further support in favor of the interference-based forgetting theories (Berman et al., 2009).

In conclusion, the present study supports previous evidence by showing that unpleasant stimuli do affect the maintenance of non-emotional information in WM. But, more specifically, in a series of three interrelated experiments and one re-analysis, the current study shows for the first time that pleasant distraction does not necessarily affect WM as unpleasant distraction does. A lower capacity of our executive control to reallocate cognitive resources after unpleasant distraction seems to account for its value as interference in WM.

### Conflict of interest statement

The authors declare that the research was conducted in the absence of any commercial or financial relationships that could be construed as a potential conflict of interest.
