# Supplementary material for: Emotional interference-based forgetting in short-term memory. Cognitive inhibition of pleasant but not unpleasant biologically relevant distractors
Source: Front Psychol. 2015 May 6;6:582. doi: 10.3389/fpsyg.2015.00582 (PMC4421942; doi:10.3389/fpsyg.2015.00582)
Supplement: Supplementary file 1 [file DataSheet1.DOCX]

***Supplementary Material***

**Emotional interference-based forgetting in short-term memory. Cognitive inhibition of pleasant but not unpleasant biologically relevant distractors**

**Javier García-Pacios ^1,2^*, David Del Río^2,3^, Dolores Villalobos^1^, José María Ruiz-Vargas^4^, Fernando Maestú^2,3^**

^1^Department of Psychology. Camilo José Cela University, Madrid, Spain

^2^Laboratory of Cognitive and Computational Neuroscience. Center for Biomedical Technology (Technical University of Madrid and Complutense University of Madrid), Madrid, Spain

^3^Department of Basic Psychology II, Complutense University of Madrid, Madrid, Spain

^4^Department of Basic Psychology, Autónoma University of Madrid, Madrid, Spain

*** Correspondence:** Javier García-Pacios, Department of Psychology. Camilo José Cela University, Madrid. C/ Castillo de Alarcón, 49, Urb.Villafrancadel Castillo. 28692, Madrid, Spain.

javier.garciapacios@ctb.upm.es

1. **IAPS scenes**

## Experiment 1

Pleasant: 1722, 2058, 2150, 2345, 2346, 4520, 4599, 4607, 4608, 4623, 4640, 4641, 4660, 4689, 5270, 5470, 5600, 5629, 5910, 7260, 7330, 7570, 8080, 8180, 8185, 8190, 8200, 8300, 8370, 8400.

Neutral: 2038, 2206, 2381, 2393, 2440, 2870, 5120, 5731, 7000, 7020, 7034, 7038, 7040, 7052, 7055, 7056, 7080, 7100, 7110, 7150, 7160, 7175, 7224, 7233, 7234, 7235, 7490, 7547, 7705, 7950.

Unpleasant: 1050, 1052, 1201, 1300, 1525, 2683, 2691, 2730, 2811, 2981, 3030, 3053, 3063, 3064, 3068, 3069, 3071, 3100, 3110, 3120, 3140, 3150, 3266, 3500, 3530, 6021, 6022, 6210, 9160, 9921.

## Experiment 2, 3 and 4

Pleasant: 4520, 4599, 4607, 4608, 4623, 4640, 4641, 4660, 4689, 5270, 5470, 5621, 5629, 5910, 7570, 8030, 8034, 8080, 8170, 8180, 8185, 8190, 8200, 8300, 8370, 8400, 8470, 8490, 8499, 8501.

Neutral: 2038, 2206, 2381, 2393, 2440, 2870, 5120, 5731, 7000, 7020, 7034, 7038, 7040, 7052, 7055, 7056, 7080, 7100, 7110, 7150, 7160, 7175, 7224, 7233, 7234, 7235, 7490, 7547, 7705, 7950.

Unpleasant: 1050, 1052, 1201, 1300, 1525, 2683, 2691, 2730, 2981, 3030, 3063, 3064, 3068, 3100, 3110, 3120, 3140, 3150, 3266, 3530, 6021, 6022, 6210, 9042, 9160, 9253, 9265, 9420, 9428, 9921.

1. **D’ values in Experiment 1, 2 , 3 and 4**

| Condition | Experiment 1 | Experiment 2 | Experiment 3 |
| --- | --- | --- | --- |
| No distraction |  | 0,95 (0,20) | 0,91 (0,16) |
| Pleasant | 0,83 (0,15) | 0,86 (0,18) | 0,82 (0,16) |
| Neutral | 0,85 (0,16) | 0,88 (0,19) | 0,81 (0,14) |
| Unpleasant | 0,76 (0,11) | 0,79 (0,17) | 0,76 (0,13) |

Standard deviations are showed in parenthesis.

1. **D’ values in Experiment 4**

| Condition | All participants | | Random down-sampling | |
| --- | --- | --- | --- | --- |
|  | Balanced | Unbalanced | Balanced | Unbalanced |
| No distraction | 0,91 (0,16) | 0,94 (0,20) | 0,91 (0,16) | 0,93 (0,21) |
| Pleasant | 0,82 (0,17) | 0,86 (0,18) | 0,82 (0,17) | 0,85 (0,19) |
| Neutral | 0,82 (0,19) | 0,88 (0,18) | 0,82 (0,19) | 0,86 (0,18) |
| Unpleasant | 0,77 (0,17) | 0,78 (0,16) | 0,77 (0,17) | 0,78 (0,17) |

Standard deviations are showed in parenthesis.

# Supplementary Results for Experiment 4

## Alternative random down-sampling A

| Condition | Corrected recognition scores | | *d’* values | | Reaction time | |
| --- | --- | --- | --- | --- | --- | --- |
|  | Balanced | Unbalanced | Balanced | Unbalanced | Balanced | Unbalanced |
| No distraction | 0,73 (0,14) | 0,76 (0,16) | 0,91 (0,16) | 0,95 (0,21) | 882,21 (113,74) | 902,37 (119,94) |
| Pleasant | 0,62 (0,17) | 0,67 (0,15) | 0,82 (0,17) | 0,87 (0,18) | 881,12 (96,92) | 888,28 (95,29) |
| Neutral | 0,62 (0,21) | 0,65 (0,18) | 0,82 (0,19) | 0,88 (0,19) | 881,66 (112,30) | 892,75 (100,93) |
| Unpleasant | 0,52 (0,17) | 0,52 (0,17) | 0,77 (0,17) | 0,79 (0,16) | 893,76 (99,94) | 906,88 (103,89) |

Standard deviations are showed in parenthesis.

## Accuracy based on corrected recognition scores

Effect of Condition: F(3, 42)=24.16, *p<*.0001, η^2^=.63; no-distraction > pleasant (*p>*.0001), neutral (*p=*.0001) and unpleasant (*p<*.0001); pleasant > unpleasant (*p<*.0001); neutral > unpleasant (*p<*.001); pleasant = neutral (*p>*.1)]

Effect of Group: F(1, 44)=0.96, *p>*.1, η^2^=.02

Effect of Interaction: F(1, 61)=0.79, *p>*.1, η^2^=.05

## Accuracy based on *d’* values

Effect of Condition: F(3, 42)=19.23, *p<*.0001, η^2^=.57; no-distraction > pleasant (*p>*.0001), neutral (*p<*.005) and unpleasant (*p<*.0001); pleasant > unpleasant (*p<*.0001); neutral > unpleasant (*p=*.001); pleasant = neutral (*p>*.1)]

Effect of Group: F(1, 44)=0.48, *p>*.1, η^2^=.01

Effect of Interaction: F(1, 61)=0.47, *p>*.1, η^2^=.03

## Reaction times

Effect of Condition: F(3, 42)=0.75, *p>*.1, η^2^=.05

Effect of Group: F(1, 44)=0.04, *p>*.1, η^2^=.006

Effect of Interaction: F(1, 61)=0.04, *p>*.1, η^2^=.003

## Subjective emotional ratings

|  | Balanced Group | | Unbalanced Group | |
| --- | --- | --- | --- | --- |
| Condition | Subjective Valence | Subjective Arousal | Subjective Valence | Subjective Arousal |
| Pleasant | 7,40 (0,97) | 5,88 (1,90) | 7,14 (0,80) | 5,35 (0,94) |
| Neutral | 4,99 (0,44) | 2,87 (1,42) | 5,08 (0,58) | 2,34 (1,19) |
| Unpleasant | 2,77 (1,14) | 6,07 (1,80) | 2,26 (0,60) | 7,00 (0,82) |

Standard deviations are showed in parenthesis.

## Alternative random down-sampling B

| Condition | Corrected recognition scores | | *d’* values | | Reaction time | |
| --- | --- | --- | --- | --- | --- | --- |
|  | Balanced | Unbalanced | Balanced | Unbalanced | Balanced | Unbalanced |
| No distraction | 0,73 (0,14) | 0,76 (0,12) | 0,91 (0,16) | 0,95 (0,16) | 882,21 (113,74) | 904,38 (120,09) |
| Pleasant | 0,62 (0,17) | 0,65 (0,16) | 0,82 (0,17) | 0,84 (0,14) | 881,12 (96,92) | 884,75 (83,62) |
| Neutral | 0,62 (0,21) | 0,65 (0,16) | 0,82 (0,19) | 0,87 (0,17) | 881,66 (112,30) | 898,30 (94,79) |
| Unpleasant | 0,52 (0,17) | 0,49 (0,18) | 0,77 (0,17) | 0,77 (0,14) | 893,76 (99,94) | 908,28 (110,98) |

Standard deviations are showed in parenthesis.

## Accuracy based on corrected recognition scores

Effect of Condition: F(3, 42)=28.05, *p<*.0001, η^2^=.66; no-distraction > pleasant (*p>*.0001), neutral (*p<*.0001) and unpleasant (*p<*.0001); pleasant > unpleasant (*p<*.0001); neutral > unpleasant (*p<*.0001); pleasant = neutral (*p>*.1)]

Effect of Group: F(1, 44)=0.20, *p>*.1, η^2^=.005

Effect of Interaction: F(1, 61)=0.50, *p>*.1, η^2^=.03

## Accuracy based on *d’* values

Effect of Condition: F(3, 42)=20.23, *p<*.0001, η^2^=.59; no-distraction > pleasant (*p>*.0001), neutral (*p<*.0001) and unpleasant (*p<*.0001); pleasant > unpleasant (*p<*.0001); neutral > unpleasant (*p=*.0001); pleasant = neutral (*p>*.1)]

Effect of Group: F(1, 44)=0.51, *p>*.1, η^2^=.01

Effect of Interaction: F(1, 61)=0.74, *p>*.1, η^2^=.05

## Reaction times

Effect of Condition: F(3, 42)=1.14, *p>*.1, η^2^=.07

Effect of Group: F(1, 44)=0.31, *p>*.1, η^2^=.007

Effect of Interaction: F(1, 61)=0.21, *p>*.1, η^2^=.01

## Subjective emotional ratings

|  | Balanced Group | | Unbalanced Group | |
| --- | --- | --- | --- | --- |
| Condition | Subjective Valence | Subjective Arousal | Subjective Valence | Subjective Arousal |
| Pleasant | 7,40 (0,97) | 5,88 (1,90) | 7,14 (0,86) | 5,55 (0,86) |
| Neutral | 4,99 (0,44) | 2,87 (1,42) | 5,06 (0,58) | 2,50 (1,22 |
| Unpleasant | 2,77 (1,14) | 6,07 (1,80) | 2,22 (0,67) | 7,06 (0,81) |

Standard deviations are showed in parenthesis.

## Alternative random down-sampling C

| Condition | Corrected recognition scores | | *d’* values | | Reaction time | |
| --- | --- | --- | --- | --- | --- | --- |
|  | Balanced | Unbalanced | Balanced | Unbalanced | Balanced | Unbalanced |
| No distraction | 0,73 (0,14) | 0,74 (0,17) | 0,91 (0,16) | 0,92 (0,19) | 882,21 (113,74) | 917,21 (146,51) |
| Pleasant | 0,62 (0,17) | 0,68 (0,14) | 0,82 (0,17) | 0,87 (0,15) | 881,12 (96,92) | 907,33 (91,63) |
| Neutral | 0,62 (0,21) | 0,69 (0,16) | 0,82 (0,19) | 0,89 (0,16) | 881,66 (112,30) | 905,30 (84,12) |
| Unpleasant | 0,52 (0,17) | 0,52 (0,19) | 0,77 (0,17) | 0,78 (0,16) | 893,76 (99,94) | 920,28 (96,11) |

Standard deviations are showed in parenthesis.

## Accuracy based on corrected recognition scores

Effect of Condition: F(3, 42)=22.84, *p<*.0001, η^2^=.62; no-distraction > pleasant (*p=*.001), neutral (*p<*.01) and unpleasant (*p<*.0001); pleasant > unpleasant (*p<*.0001); neutral > unpleasant (*p<*.0001); pleasant = neutral (*p>*.1)]

Effect of Group: F(1, 44)=0.69, *p>*.1, η^2^=.01

Effect of Interaction: F(1, 61)=0.91, *p>*.1, η^2^=.06

## Accuracy based on *d’* values

Effect of Condition: F(3, 42)=19.51, *p<*.0001, η^2^=.58; no-distraction > pleasant (*p=*.001), neutral (*p<*.01) and unpleasant (*p<*.0001); pleasant > unpleasant (*p<*.0001); neutral > unpleasant (*p<*.0001); pleasant = neutral (*p>*.1)]

Effect of Group: F(1, 44)=0.74, *p>*.1, η^2^=.01

Effect of Interaction: F(1, 61)=1.13, *p>*.1, η^2^=.07

## Reaction times

Effect of Condition: F(3, 42)=0.61, *p>*.1, η^2^=.04

Effect of Group: F(1, 44)=1.21, *p>*.1, η^2^=.02

Effect of Interaction: F(1, 61)=0.03, *p>*.1, η^2^=.002

## Subjective emotional ratings

|  | Balanced Group | | Unbalanced Group | |
| --- | --- | --- | --- | --- |
| Condition | Subjective Valence | Subjective Arousal | Subjective Valence | Subjective Arousal |
| Pleasant | 7,40 (0,97) | 5,88 (1,90) | 7,21 (0,86) | 5,33 (0,98) |
| Neutral | 4,99 (0,44) | 2,87 (1,42) | 5,11 (0,60) | 2,31 (1,14) |
| Unpleasant | 2,77 (1,14) | 6,07 (1,80) | 2,16 (0,64) | 6,68 (1,21) |

Standard deviations are showed in parenthesis.
